# Development of Continuous Additive-Controlled MSMPR Crystallization by DoE-Based Batch Experiments

**DOI:** 10.1021/acs.iecr.4c01933

**Published:** 2024-07-23

**Authors:** György Nimród Stoffán, Zsolt Lőrincz, Éva Pusztai, Lajos Madarász, Kornélia Tacsi, György Marosi, Hajnalka Pataki

**Affiliations:** † Department of Organic Chemistry and Technology, Faculty of Chemical Technology and Biotechnology, Budapest University of Technology and Economics, Műegyetem rkp. 3, Budapest 1111, Hungary; ‡ Department of Chemical and Environmental Process Engineering, Faculty of Chemical Technology and Biotechnology, Budapest University of Technology and Economics, Műegyetem rkp. 3, Budapest 1111, Hungary

## Abstract

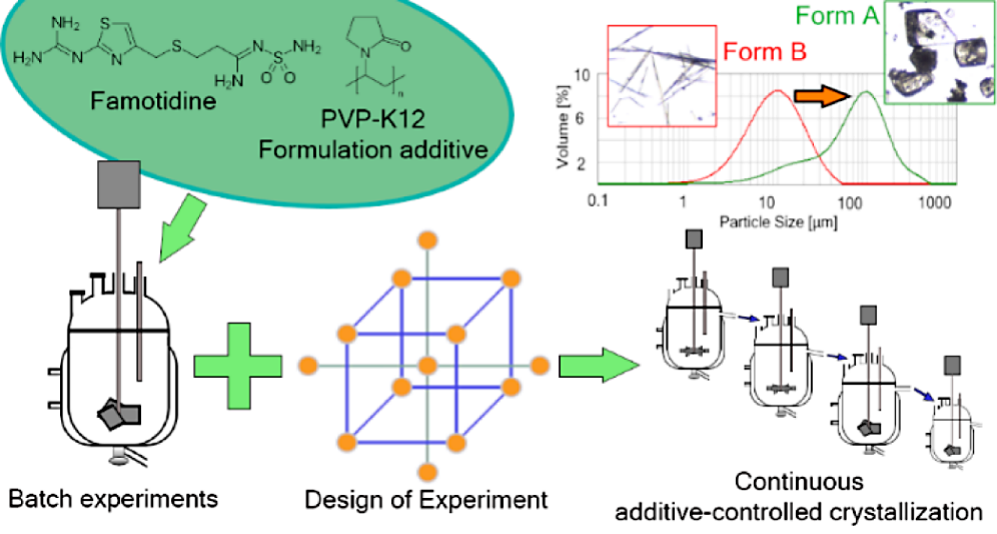

Additive-controlled crystallization is a promising method to improve crystal morphology and produce solid drug particles with the desired technological and pharmacological properties. However, its adaptation to continuous operation is a hardly researched area. Accordingly, in this work, we aimed to come up with a methodology that provides the systematic and fast development of a continuous three-stage MSMPR cascade crystallizer. For that, a cooling crystallization of famotidine (FMT) from water, in the presence of a formulation additive, poly(vinylpyrrolidone) (PVP-K12), was developed. Process parameters with a significant impact on product quality and quantity were examined in batch mode through a 2^4–1^ fractional factorial design for the implementation of additive-controlled continuous crystallization. These batch experiments represented one residence time of the continuous system. Based on the statistical analysis, the residence time (RT) had the highest effect on yield, while the polymer amount was critical from the product polymorphism, crystal size, and flowability points of view. The values of critical process parameters in continuous operation were fixed according to the batch results. Two continuous cooling crystallization experiments were carried out, one with 1.25 w/w_FMT_% PVP-K12 and one with no additive. A mixture of FMT polymorphs (Form A and Form B) crystallized without the additive through five residence times (>6.5 h) with 70.8% overall yield. On the other hand, the additive-controlled continuous experiment resulted pure and homogeneous Form A product with excellent flowability. The system could be operated for >6.5 h without clogging with a 71.1% overall yield and a 4-fold improvement in productivity compared to its batch equivalent.

## Introduction

In the pharmaceutical industry, crystallization is the primary technological step to separate, purify, and control the crystalline properties of the drug product.^1^ However, in a relevant number of cases, traditional crystallization methods (cooling, reactive, antisolvent, etc.) cannot provide the expected polymorphism, morphology, and, by that, pharmacological and technological qualities of the solid active pharmaceutical ingredient (API). Furthermore, to fine-tune the physical properties of the drug (crystal habit, size, and crystal size distribution), other downstream processes (e.g., milling, mixing, granulation, etc.) are introduced in the production technology to meet the quality expectations. This can lead to a longer, and more costly production process,^2^ and can also potentially induce unwanted polymorphic transformation. Additive-controlled crystallization is based on heterogeneous nucleation, in which a carefully selected material is added to the crystallization media to alter the solid phase’s physical properties in the targeted way. Every compound, chemically different from the substance to be crystallized, which is purposefully added to the crystallization media and influences the subprocesses of the crystallization mechanism (nucleation, crystal growth, aggregation, etc.) can be considered as an additive.^3,4^ Therefore, the chemical and functional diversity of additives can be seemingly endless. The most common molecule types cover different polymers (e.g., excipients), surfactants,^5,6^ predesigned small molecules called tailor-made additives,^7,8^ small inorganic and organic molecules,^9,10^ self-assembling monolayers (SAMs)^11,12^ and so on. Based on their function, additives can be categorized as promoters, which initiate the process of crystallization, and inhibitors, which delay nucleation. Crystallization additives can also be divided into at least two more groups according to their role in nucleation and crystal growth, which is closely related to their solubility in the crystallization medium.^4,13,14^ Insoluble additives can serve as potential nucleation surfaces, and their surface properties, such as porosity, pore size, and coarseness, besides their functional groups, play a major role in the nucleation and crystal growth of the target molecule.^15,16^ Soluble additives can change the solubility of the API by forming secondary bonds with the solvent, API, or themselves. They can significantly widen the metastable zone, elongate induction time, or completely stabilize the supersaturated solution.^17^ Overall, additive-controlled crystallization has already been successfully employed as a technological tool for polymorphic,^18,19^ size and habit control of APIs,^8,14,20,21^ as well as for process stability enhancement^22^ and simplification of downstream formulation procedure.^23,24^ Most of these additives are well-known formulation additives with low or no toxic effects on humans; therefore, the industrial application of such crystallization technologies should give no rise for regulatory holdbacks. All at the same, there are numerous examples of additive-controlled crystallization which are dominantly performed in batch mode, and only a few publications discuss the adaptation of continuous technologies. In turn, the advantages of continuous technologies, such as improved productivity, constant product quality, and technological flexibility, make them more economical compared to their batch equivalents.^25,26^ In addition, these benefits fit well with the idea of additive-controlled crystallization to simplify downstream formulation by targeted morphology alteration to make solid API production more efficient and economical.

Continuous crystallization is a well-established research field with two basic operational implementations, which are the mixed suspension mixed product removal (MSMPR) crystallizers^27,28^ and different tubular crystallizers (TC).^29−34^ The assembling of more MSMPR units into a cascade crystallizer system^35^ or the connection of different types of continuous crystallizers^36−40^ allows even more possibilities to control crystallization on its subprocess levels. The elevated performance of merging continuous and additive-controlled crystallization is demonstrated through the following examples from the literature. Powell et al. focused on the difficulties of paracetamol Form I crystallization in a single-stage MSMPR caused by fouling and encrustation on PAT (process analytical technologies) probes and the vessel wall.^22^ When hydroxypropyl methylcellulose (HPMC) was added to the crystallization media, not only could encrustation and fouling be avoided, but steady-state operation was achieved more rapidly. Tacsi et al. investigated the continuous antisolvent crystallization of acetylsalicylic acid in the presence of PVP in an integrated TC-MSMPR system.^24^ The small crystals produced in the first sonicated TC aggregated in the second MSMPR stage, resulting in simultaneously fast dissolution characteristics and improved technological properties. Testa et al. successfully developed an evaporative-cooling-MSMPR system for the continuous heterogeneous crystallization of paracetamol.^41^ They used poly(vinyl alcohol) (PVA) as a crystallization surface, and the crystallizer could be adopted to an end-to-end system. Hu et al. also tested different excipients (PVA, PVP, etc.) to alter the morphology of carbamazepine.^42^ In their work, they identified the significant process parameters which determined the polymorphism and overall morphology of the API in a single-stage MSMPR.

Based on the few continuous additive-controlled crystallization examples, the literature is still short of systematic workflows for developing and optimizing these technologies. Design of Experiment (DoE) is an effective and simple method to identify critical process parameters (CPPs), which provides an opportunity to gain a deeper understanding of the process by exploring the parameter dependencies of product properties.^43,44^ Besides DoE, the incorporation of batch results and observations is another feasible solution for the development of continuous technologies.^45−47^ In contrast, to the best knowledge of the authors, there is no case study present in the relevant literature, which utilizes the batch results and the systematic framework of DoE at the same time to develop continuous additive-controlled crystallization technologies. However, previously in other fields of science, a similar process development methodology was used to transfer batch results to continuous technology for flow syntheses^48,49^ and chromatography.^50^

Both additive-controlled and continuous crystallization are well-established fields and are powerful tools in the efficient, targeted crystal engineering of APIs. Despite their own, but complementary, advantages, the two technologies are seldom combined. Accordingly, the results of DoE-based small-scale batch experiments could contribute to the development of more efficient continuous additive-controlled crystallization technologies. The famotidine (FMT) model drug we have chosen, is a histamine H_2_-receptor antagonist, which inhibits the secretion of gastric acid. FMT has three polymorphs, namely, Forms A, B, and C, and an amorphous form; however, the literature only focuses on Form A and Form B, which are conformational polymorphs. Form A is the thermodynamically stable crystal form, which exhibits isometric crystal habit, and Form B is the kinetically preferred one, crystallizing in a needlelike structure, while Form C exhibits the least crystalline properties and is closer to amorphous form.^51−53^ The outcome of cooling crystallization of FMT is mostly dependent on the applied solvent, initial concentration, cooling rate, and seeding conditions.^54^ The polarity and the ability of the solvent to establish strong hydrogen bonds with FMT play a crucial role in the nucleation of Form B. Strong intramolecular hydrogen bonds established with water molecules stabilize the folded conformation of FMT Form B, while less polar solvents, such as methanol and acetonitrile, aid the nucleation of Form A. At high FMT concentrations, predominantly Form B is formed. Nevertheless, at the same conditions, when the cooling rate is not high enough, at low levels of supersaturation, Form A can nucleate as well. Due to the lower solubility of Form A, at certain concentration and temperature combinations, the supersaturation of Form A is higher than that of Form B. Besides crystallization, the polymorphic transformation of FMT Form B to Form A can be solvent-mediated as well. In addition, Ravouru et al. report the morphology changes achieved with PVP-assisted crystallization of FMT, which resulted better pharmacological and therapeutic properties.^55^ These crystals had good dissolution profile, significant antioxidant activity, increased stability, and antiulcer activity.^55^ The authors refer to the crystallized products as different polymorphs; however, they conclude that compared to the initial FMT crystals, the added PVP altered the habit of the API. In summary, the crystallization of FMT is well described in the literature, but no previous attempt was made to implement it in continuous mode.

In this work, we aimed to develop a three-stage MSMPR crystallizer for the additive-controlled cooling crystallization of FMT from water with PVP. Our aim was to develop a methodology of systematic examination of CPPs on critical quality attributes (CQAs) and thus establish the process parameters for its continuous crystallization technology. To achieve this, a series of fast, small-scale, and controlled batch experiments were conducted, avoiding time- and material-consuming continuous experiments to represent one residence time of the continuous crystallizer in question. The experiments were based on a 2^4–1^ fractional factorial design to identify significant factors on FMT polymorphism, morphology, powder flowability, yield, and continuous operability. Later, these results could be subjected to further optimization of the developed continuous procedures.

## Experimental Section

### Materials

Famotidine (>99.9% purity, Form B polymorph) was donated by Gedeon Richter Plc., PVP-K12 (*M*_w_ = 5000 Da) was obtained from International Specialty Products (ISP), and deionized water was produced in the laboratory.

### Methods

#### Batch Experiments

**Figure 1:**
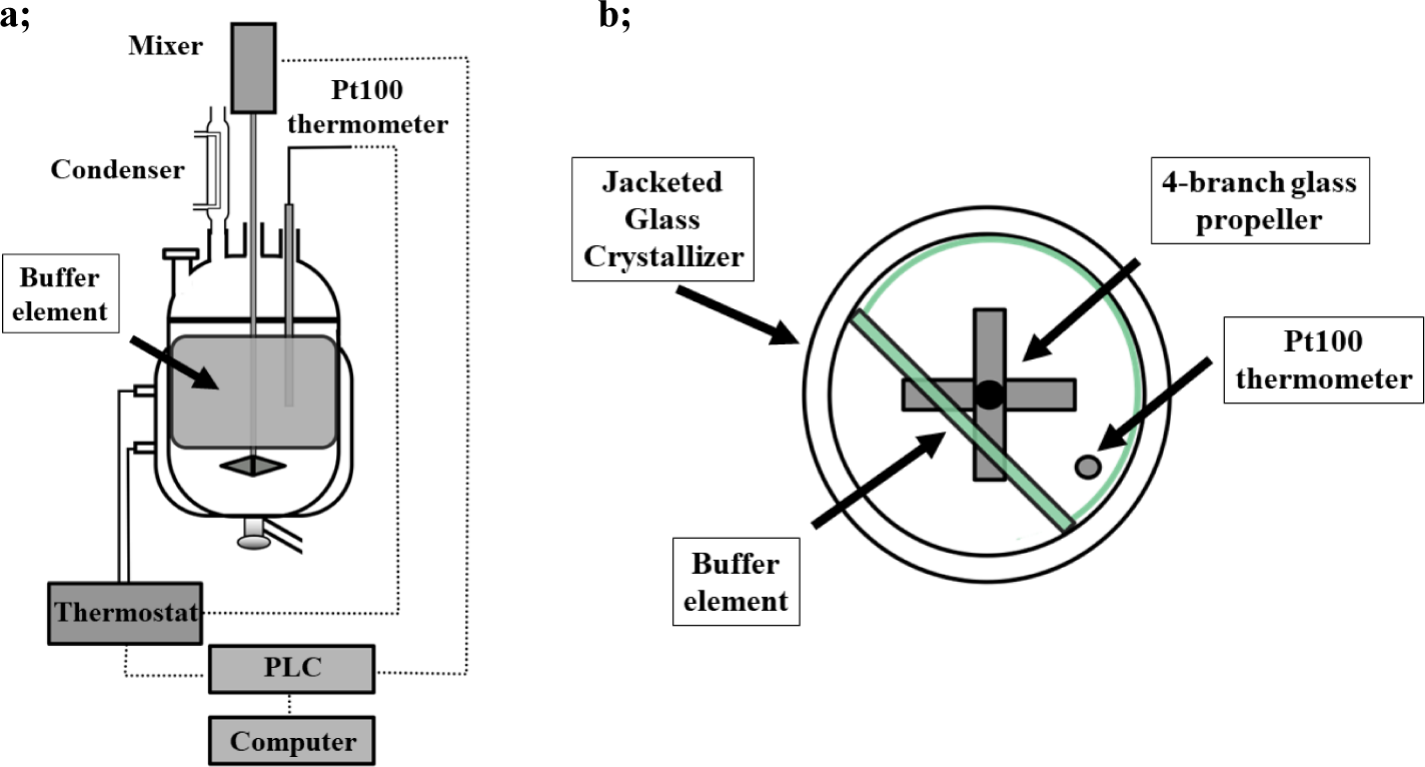
Schematic images representing the batch crystallizer (a) and the 3D printed buffer element in its position (b).

Batch crystallization experiments were conducted in a 150 mL double-walled glass crystallizer. A 4-branch glass propeller stirrer, a mixer (Eurostar type, IKA), a glass condenser, and a Pt100 resistance thermometer were connected to the crystallizer. Silicone oil flowed in the jacketed chamber, and its temperature was controlled by an in-house developed monofluidic thermostat system. The process parameters (temperature and mixing rate) were set using a STARDOM-type programmable logic controller (PLC, Yokogawa Hungaria Ltd., Hungary) via Logic Designer software. A custom-designed, 3D-printed buffer element was placed inside the crystallizer to improve the mixing efficiency and later enhance homogeneous product removal from the overflowing continuous crystallizer. The buffer element (103 × 52 mm) was made of poly(lactic acid) (PLA) filament with a circular support formed on top of the reactor (see Figure 1).

**Figure 2:**
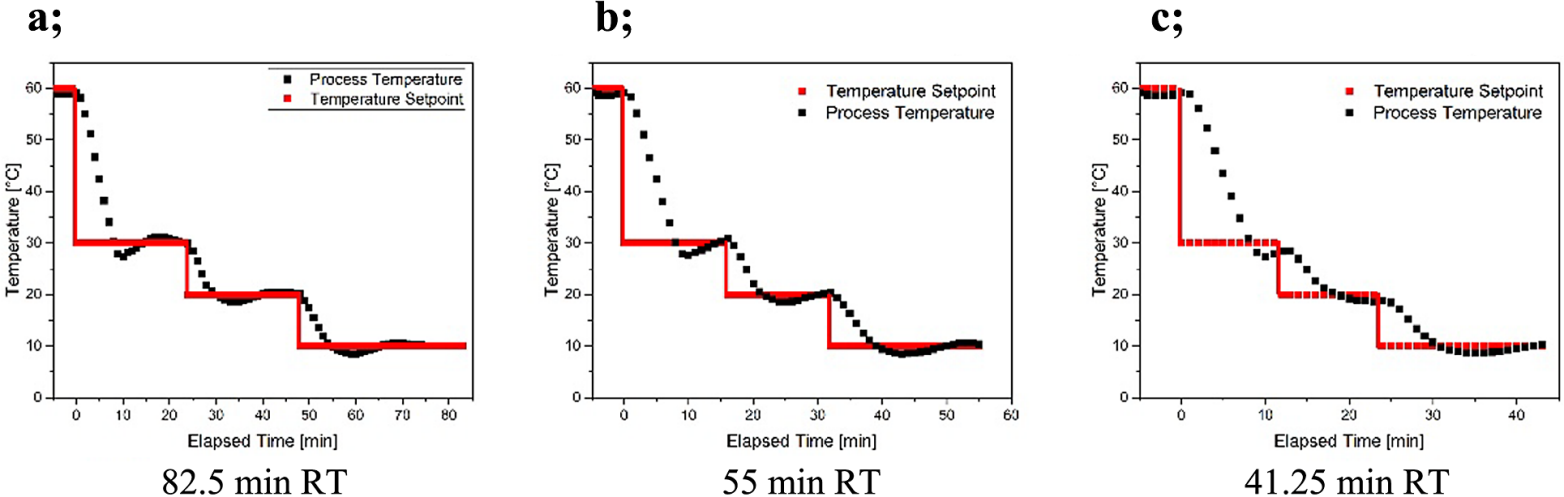
Different set point and actual temperature profiles of the batch experiments.

In each batch experiment, 1.05 g of FMT Form B was dissolved in 150 mL of water solvent. It was a 0.007 g/mL FMT solution, where the saturated concentration of FMT Form B was at 55 °C.^53^ At the beginning of the experiments, the required amounts of PVP (0 w/w_FMT_%, 0 g or 1.25 w/w_FMT_%, 0.0131 g or 2.5 w/w_FMT_%, 0.0263 g) were added to the FMT suspension in the crystallizer, and then the crystallization program with the appropriate temperature profile was started along with constant mixing (200, 300, or 400 rpm). The suspension was quickly heated to 60 °C and held at a constant temperature for 5 min to ensure complete dissolution and stabilize the temperature. The cooling profile is designed to match as closely as possible to continuous cooling crystallization in a three-stage MSMPR crystallizer over one total residence time (*RT*). The MSMPR cascade consisted of three 250 mL and one 100 mL MSMPR units, where in the last two units, the same temperature (10 °C) was set to match the three temperature stages. The residence times in the continuous crystallizer would be provided by the feeding rate (*FR*) of the feed pump(s) (10, 15, or 20 mL/min). Therefore, the effect of three different isothermal temperature stages 30, 20, and 10 °C and three different total residence times 41.25, 55, and 82.5 min were studied in batch experiments. At each temperature step, the appropriate residence time, characterizing one crystallizer stage, was applied with cooling as fast as possible between these isotherm stages. The residence time of each stage is calculated as the fraction of the filling volume of the given MSMPR unit and the set flow rate (see eq 1). Accordingly, the applied temperature profiles of batch crystallization are shown in Figure 2.
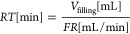
1

After crystallization, the suspension was filtered through a G3 porosity glass filter (pore size of 16–40 μm) with a membrane pump and air-dried for 3 days. No washing was applied during the filtration. Subsequently, the yield (*y* [%]) was calculated from the weighed mass according to eq 2, where *m*_gross_ is the mass of the air-dried sample and the glass filter, *m*_tare_ is the previously measured mass of the glass filter, and *m*_Form_B_ is the measured mass of FMT Form B into the crystallizer. The chemical composition of the product was determined by mapping with Raman spectroscopy. The physical properties of the crystals were analyzed by optical microscopy, the laser diffraction method, and powder flowability analysis.
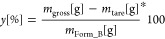
2

The batch experiments were carried out based on a 2^4–1^ fractional factorial design to explore the effects of the added PVP-K12 (*p*_PVP-K12_; 0–2.5 w/w_FMT_%), residence time (RT or its continuous equivalent flow rate—*FR*; 10 mL/min, 82.5 min and 20 mL/min, 41.25 min), buffer element (BE; yes or no) and stirring rate (*f*; 200–400 rpm) on yield (*y*[%]), and product quality (polymorphism, crystal size, habit, agglomeration, powder flowability) (see Table 1).

**Table 1:** Applied Experimental Settings of the 2^4–1^ Fractional Factorial Design

	lower level (−)	center point	upper level (+)
*p*_PVP-K12_[w/w_FMT_%]	0	1.25	2.5
*f*[RPM]	200	300	400
BE[yes or no]	no	-a	yes
*RT* [min]^b^(*FR* [mL/min])^c^	82.5 (10)	55 (15)	41.25 (20)

a BE is a categorical factor; therefore, center point is not interpretable.

b Residence time values to be set in batch experiment representing 1 RT in continuous operation.

c Flow rate values to be set in continuous crystallization.

A total of 8 different corner setting points and 4 center point experiments were conducted. The experiments were statistically analyzed by using TIBCO Statistica 14.0.0.15 software. For all statistical tests, α = 0.05 significant level was used.

### Continuous MSMPR Experiments

**Figure 3:**
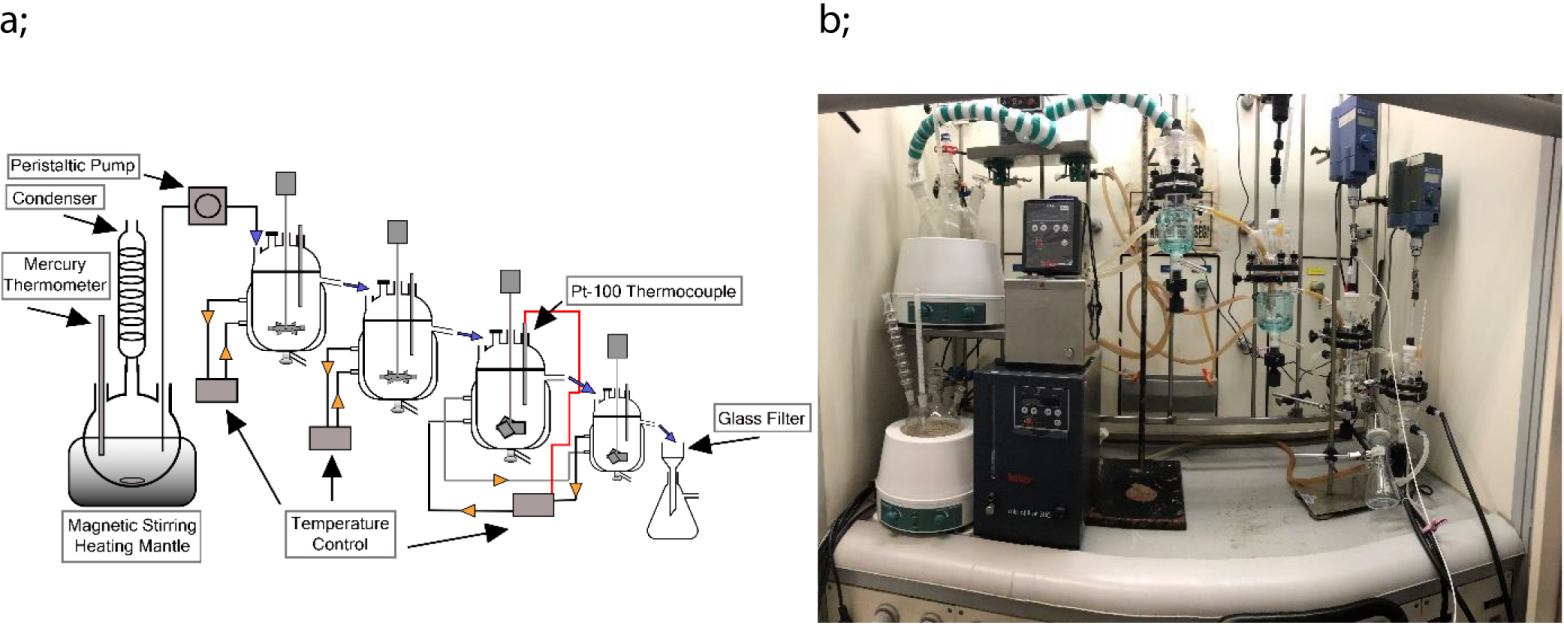
Schematic (a) and photographic (b) illustrations of the continuous crystallizer system.


Continuous crystallization was executed in a three-stage MSMPR cascade crystallizer system (30–20–10 °C) consisting of four MSMPR units supplemented with a feed round flask (2 L) and a peristaltic pump (Figure 3). The feed round flask was complemented with a condenser, a thermometer, and a 98-II-B type (Faithful Instrument, China) magnetic stirring heating mantle to reach the initial solution temperature (60 °C). The heated solution was transferred to the first MSMPR unit with a peristaltic pump (Pump *p*-1, Cytiva, USA), capable of a maximum 10 mL/min flow rate, via a PTFE tube (ID: 0.4 mm). The PTFE tube was insulated to prevent a drastic temperature drop and early nucleation. The solution was fed to the first MSMPR at a constant flow rate (10 mL/min). All four MSMPRs (Schmizo, Switzerland) were jacketed glass crystallizers equipped with an overflow tube. The nominal volumes of the MSMPRs were 250 mL for the first 3 and 100 mL for the last MSMPR unit. The inner diameter of the overflow tube was 7.0 mm for each MSMPR. Their length and tilt angle were 5.5 cm and 10°, respectively, for the first, second, and fourth MSMPRs in row, and the third MSMPR had a 3.5 cm long horizontal overflow tube. Besides, all overflow tubes had rubber tube extensions, which were submerged into the suspensions in the crystallizers for efficient mixing. In the first and second MSMPRs, proper mixing was executed with 6-blade radial impellers (horizontal overall dimension: 35 mm) connected to R20 overhead stirrers (CAT Scientific, Germany), while in the third and fourth MSMPR units, it was accomplished with 3-blade marine impellers (horizontal overall dimension: 35 mm) connected to Eurostar-type mixers (IKA, Germany). Each impeller was coated in PTFE. The desired isotherm temperature steps were set through controlling the jacket temperature of the MSMPRs. The first MSMPR (30 °C) was connected to a Kiss 202C (Huber, Germany) circulation thermostat, and the second MSMPR (20 °C) was connected to an OLÉ 300 (Huber, Germany) minichiller. Both the thermostat and the minichiller were filled with ethylene-glycol as thermotic medium. The last isotherm step (10 °C) was facilitated in the third and fourth MSMPR units, which were commonly connected to a Ministat 230 (Huber, Germany) thermostat filled with silicone oil. The temperature of the slurry was measured with mercury thermometers in the first, second, and fourth MSMPR units and with a Pt-100 thermocouple connected to the Ministat 230 thermostat in the third unit. The product was collected through the overflow tube of the fourth MSMPR on a G3 pore-size glass filter under a constant vacuum. At the end of every residence time, samples were collected separately to be analyzed and characterize the system at each *RT*. No washing was applied during filtration, and the samples were air-dried for 3 days. The yield (*y*[%]) was calculated from the total amount of solid product retrieved *∑m*_retrieved_ [g] and the total input of FMT during crystallization, where *t*_elapsed_ [min] is the elapsed time until the end of the experiment, *FR* is the flow rate (10 mL/min), and *c*_solution_ is the concentration of the feed solution (0.007 g/mL) (see eq 3.). The productivity (*p*[g/h]) of the crystallization processes was defined as the fraction of the total amount of solid product retrieved *∑m*_retrieved_ [g] and the elapsed time (*t*_elapsed_ [h]) (see eq 4).

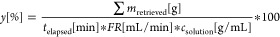
3


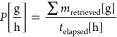
4

Two continuous crystallization experiments were investigated in the presence of 1.25 w/w_FMT_% PVP-K12 related to the mass of FMT, and without any additive. Other experimental settings were determined based on the results of the batch experiments. Thus, a 10 mL/min flow rate of the feed solution, 300 rpm mixing rate in each MSMPR, and no buffer elements were generally applied.

For continuous crystallization experiments, the following startup strategy was applied. In advance, 2000 mL of 0.007 g/mL aqueous FMT feed solution was prepared by heating the suspension to the initial temperature (60 °C). To avoid the time-consuming filling of the MSMPR units by the feed pump, 800 mL of 0.007 g/mL aqueous FMT solution was prepared, in a similar manner discussed above, and divided among the four MSMPR units. The solutions were then cooled to operating temperatures in each unit, e.g., 30–20–10 °C, respectively. During the experiments, the feed solution was refilled with 1000 mL of tempered solution once it decreased to half of its initial volume. The refilment during continuous crystallization was repeated as much as required.

### Analytical Methods

#### Raman Spectroscopy Measurements

The polymorphic composition of the products was determined by Raman spectroscopic mapping. The maps were recorded with a LabRAM-type Raman spectrometer (Horiba Jobin Yvon, France) equipped with a CCD detector and a 785 nm (100 mW) diode laser. The Raman maps were recorded with the following measurement settings; no filters were applied, 10× objective, 950 cm^–1^ optical grating position, 5 s spectral acquisition time per spectrum, 3 accumulation number, and 200 μm step size for a region of 2000 μm × 2000 μm around the center of the sample. The maps consisted of 121 individual measurement points. All maps were evaluated using LabSpec 5 software by the classical least-squares (CLS) method using reference spectra of pure polymorphs, namely, FMT Form A and Form B. The spectra were normalized at the whole wavenumber range, and multipoint linear baseline correction was applied. The aim of the CLS method is to generate the spectra of the sample as a linear combination of the reference spectra of pure polymorphs. The so-called spectral concentrations in percentage obtained by calculations present the ratio of the reference spectra and are proportional to the actual composition of the sample.

#### Optical Microscopic Measurements

The appearance, habit, and size of crystal products were examined with a CKX53 inverse optical microscope (Olympus, Japan) equipped with 4×, 10×, and 20× objectives. The optical microscopic images were taken with an SC180 digital 4K, UHD, 18 Mpx camera connected to the microscope using cellSensEntry 2.3 software. The samples were dispersed in a silicone oil.

#### Crystal Size and Crystal Size Distribution Measurements

The crystal size and crystal size distribution of the products were determined using a Mastersizer 2000 (Malvern Instruments, UK) connected to a Scirocco 2000 dry powder feeder (Malvern Instruments, UK). Around 500 mg of sample was used per measurement, which was dispersed with 0.5 bar overpressure. The measurement time was set to 10 s, followed by a 10 s cleaning section to ensure no particle remains in the device from the previous measurement. The crystal size distribution (CSD) was evaluated using volumetric distribution values, e.g., Dv10, Dv50, and Dv90 and the span, which is the width of the distribution calculated according to eq 5.
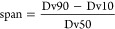
5

#### Powder Flowability Analysis

The compressibility of the experimental products was determined by using an SVM 12 (Erweka, Germany) tapped density tester. About 5 mL of solid sample was poured into a 10 mL measuring cylinder and placed on the device, which tapped the measuring cylinder for 1 min with 3 strokes/s. The flowability of the samples was characterized by calculating the Carr index according to eq 6, where *ρ*_bulk_ and *ρ*_tapped_ are the calculated densities of the sample before and after measurement, respectively (see the Supporting Information).
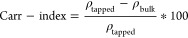
6

## Results and Discussion

### Batch Experiments

A series of batch crystallization experiments were carried out to study the effects of process parameters on the product quality and quantity. Our aim was to set up qualitative and quantitative correlations between the CPPs and CQAs (e.g., yield, polymorphism, crystal size, powder flowability, etc.). These examined process parameters included drug concentration (*c*_FMT_), polymer mass fraction (*p*_PVP-K12_), residence time (*RT*, by which flow rate—*FR* and cooling profile), initial solution temperature (*T*), stirring speed (*f*), and presence of a buffer element (BE). To reduce the number of necessary experiments for statistical analysis, some process parameters were fixed in advance (see the Supporting Information).

The values of three process parameters (*c*_FMT_, *T*, and temperature step profile) were fixed in the experimental design. The drug concentration was set to 0.007 g/mL, since applying a higher API concentration more likely caused clogging in the peristaltic feed pumps. In addition, the feed solution temperature was also fixed at 60 °C, 5 °C above the saturation temperature of the solution (55 °C). The temperature profile was set as 30–20–10 °C to represent the continuous cascade system. In order to examine the effect of PVP-K12 on drug morphology, 2.5 w/w_FMT_% of PVP-K12 was chosen as the upper limit of the experimental design, since at higher amounts of polymer, the product stuck on the crystallizer wall significantly. The mixing rate and the buffer element can influence the particle size and homogeneous product withdrawal by changing the mixing conditions; for this end, investigation of these effects was also necessary. Therefore, a 2^4–1^ fractional factorial experimental design was accomplished to simplify the full experimental design and decrease the necessary number of experimental runs. The fourth factor was introduced to the place of the *x*_1_*x*_2_*x*_3_ interaction of the corresponding 2^3^ full factorial design. Due to the nature of the applied fractional factorial design, the resolution of the design is four (*R* = IV). Therefore, the two-factor interactions confound with one another and cannot be evaluated accurately and separately (see Table 2).

**Table 2:** Confounding Effects of the Applied 2^4–1^ Fractional Factorial Design

*p*_PVP-K12_ = *f* · BE *·RT*
*f* = *p*_PVP-K12_ · BE · *RT*
BE = *p*_PVP-K12_ · *f* · *RT*
*RT* = *p*_PVP-K12_ · *f* · BE
*p*_PVP-K12_ · *f* = BE · *RT*
*p*_PVP-K12_ · BE = *f* · *RT*
*p*PVP-K12 · *RT* = *f* · BE

The above-described fractional factorial design was supplemented with four center point experiments as well. Since the presence of the buffer element is a categorical factor, meaning it is either present in or absent from the crystallizer, it has no feasible center point setting. Therefore, two types of center point experiments were carried out with *RT*, *f*, and *p*_PVP-K12_ set to their center point levels and with or without the BE. The linearity of the fitted statistical model, the variance (*Var*(*y*)), and repeatability can be investigated by supplementary center point experiments. By randomizing the experiments, the effect of the factors could be separated from the effect of the unobserved, time-varying conditions (see the Supporting Information).

The set process parameters with the achieved yield and product composition of the experiments are summarized in Table 3.

**Table 3:** Set Process Parameters of the Experiments According to the 2^4-1^ Fractional Factorial Design and Experimental Results

experiment name	randomized order	*p*_PVP-K12_ [w/w_FMT_%]	*f* [RPM]	BE [yes or no]	*RT* [min] (*FR* [mL/min])	Form A polymorph content [%]	*y* [%]
FMT_1	12	2.5	400	yes	41.25 (20)	100	69.0
FMT_2	1	2.5	400	no	82.5 (10)	100	84.0
FMT_3	7	2.5	200	yes	82.5 (10)	100	76.4
FMT_4	5	2.5	200	no	41.25 (20)	100	69.1
FMT_5	8	0	400	yes	82.5 (10)	11	82.3
FMT_6	6	0	400	no	41.25 (20)	78	68.0
FMT_7	11	0	200	yes	41.25 (20)	2	66.8
FMT_8	4	0	200	no	82.5 (10)	4	82.2
FMT_9	9	1.25	300	yes	55 (15)	100	69.6
FMT_10	10	1.25	300	yes	55 (15)	100	69.2
FMT_11	2	1.25	300	no	55 (15)	100	76.6
FMT_12	3	1.25	300	no	55 (15)	100	77.1

The yield varied between similar limits in the absence (66.8–82.3%) and presence of PVP-K12 (69.0–84.0%) under the experimental design conditions. The lowest amount of product was produced when no polymer was added to the crystallizing solution, in the presence of the buffer element, at 200 rpm mixing and a 20 mL/min flow rate (41.25 min *RT*). The highest yield was achieved using 2.5 w/w_FMT_% PVP-K12, with no buffer element present at 400 rpm and 10 mL/min (82.5 min of *RT*). On the other hand, 1.25 and 2.5 w/w_FMT_% PVP-K12 brought about the crystallization of pure Form A polymorph, while without the polymer present in the solution, a mixture of Form A and Form B arose. When no additive was used, the dominant polymorph was Form B, but Form A also appeared in varying amount. The statistical evaluation and detailed analysis of the results are discussed in the following sections.

### Statistical Analysis of Yield

**Figure 4:**
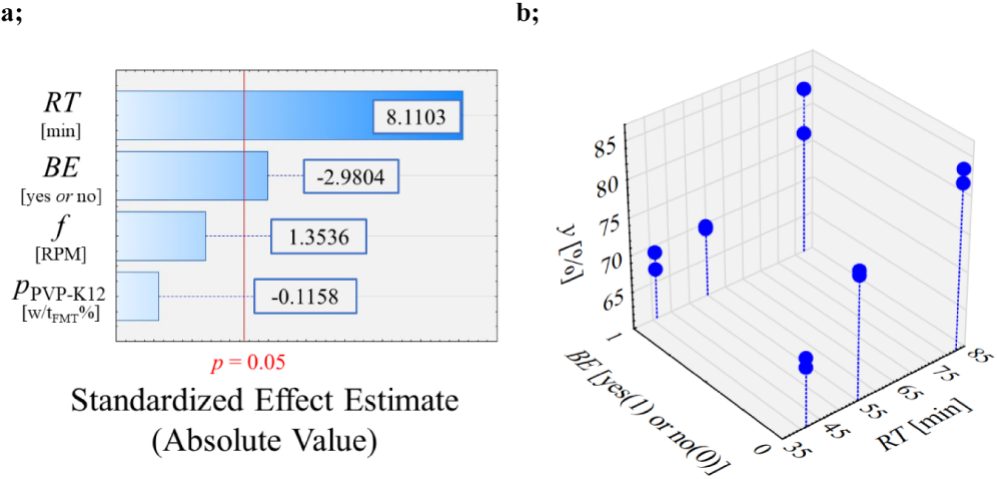
Pareto-chart of yield (a) and yield as a function of RT and BE (b).

First, a primary statistical model was fitted to investigate the effects of process parameters on the yield. The analysis of interactions of the main effects was ignored, since the applied 2^4–1^ fractional design does not allow the precise estimation of factor interactions. According to the *t*-test and Pareto chart, the *RT* (or *FR* in continuous crystallization) and BE were statistically significant (*p* < 0.05) (Table 4 and Figure 4a). Mixing rate (*f*) and, surprisingly, the presence or the amount of the added polymer (*p*_PVP-K12_) have no significant effect on yield under the examined conditions of the process. The results of the *t*-tests and the corresponding *p*-values of the analyzed factors are summarized in Table 4.

**Table 4:** Results of the Statistical Analysis of Yield^a^^b^

	*t*	*p*
**mean/intercept**	112.28	**0.0000**
*p*_PVP-K12_ [w/w_FMT_%]	–0.12	0.9111
*f*[RPM]	1.35	0.2179
**BE [yes or no]**	–2.98	**0.0205**
***RT* [min]**	8.11	**0.0001**

a *R*^2^ = 0.9161; mean-squared residual = 5.2472.

b Statistically significant factors are displayed in bold.

Plotting yield against *RT* and BE (Figure 4b), it can be stated that there is a difference of functionality, which depends only on the presence of the buffer element. When the buffer element is absent from the crystallizer, the yield is a sigmoid function of *RT*. On the other hand, yield is an exponential-like function of *RT*, when the buffer element is present. Therefore, the two cases should be handled separately and can be approximately described as in eq 7 (no BE) and eq 8 (with BE).
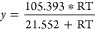
7
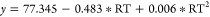
8

Accordingly, to maximize yield in continuous operation, the flow rate is recommended to be set to 10 mL/min, since it is the continuous equivalent of 82.5 min of residence time, and the usage of the buffer element is to be avoided.

### Characterization of Polymorphism

**Figure 5:**
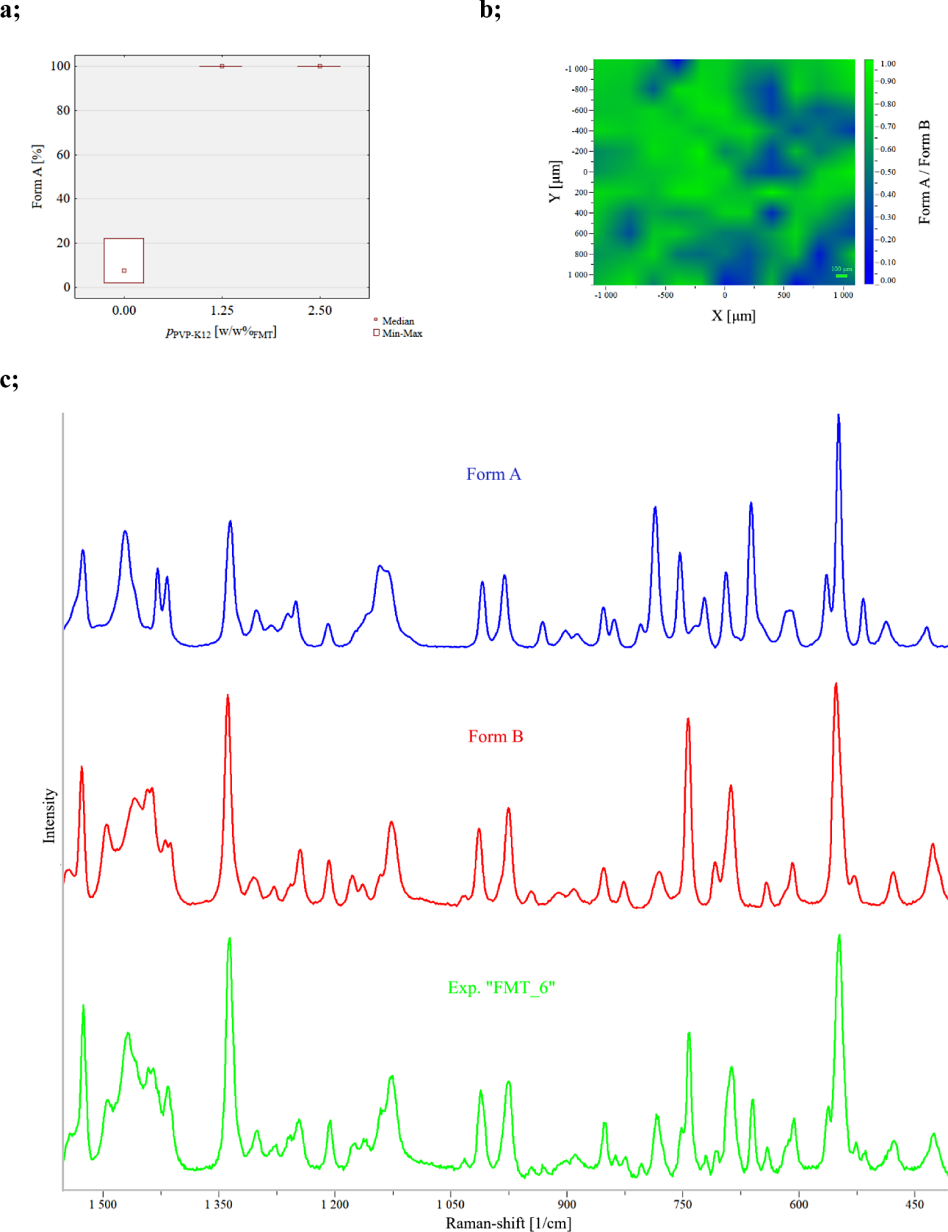
Form A quantity as a function of PVP-K12 (a), Raman map of experiment FMT_6 (b) and (c) a single spectrum from the Raman map of the reference spectra of Form A and Form B polymorphs, and experiment FMT_6.

In all cases, when PVP-K12 was dissolved in the initial solution (1.25 and 2.5 w/w_FMT_%), crystallization of the pure thermodynamically stable Form A polymorph occurred (see Figure 5a). When the crystallization of FMT was attempted without PVP-K12, a mixture of the two polymorphs, namely, Form A and the kinetically preferred Form B, was identified. The distributions of the two polymorphs in the crystalline products were homogeneous (see Figure 5b), and their ratio could be identified by CLS evaluation of the Raman maps, based on the changes in the Raman spectrum compared to the reference spectra (see Figure 5c). The polymorphic composition of these mixtures was dominated by Form B (89–98%), except one case, when the ratio of the Form A and Form B polymorphs turned up to be 78–22%, respectively. This phenomenon could be explained by the solvent-mediated polymorphic transformation of metastable Form B to the thermodynamically stable Form A in suspension. This transition could be accelerated by higher temperatures or a slight amount of Form A, although these effects were not investigated in this system by the authors, and no exact correlation could be defined statistically between the independent and dependent variables. It can be concluded that, to avoid varying polymorphic composition of the product, PVP-K12 is essential to control the nucleation of Form A polymorph. It was found that increasing the amount of the added polymer influences the size of the nascent Form A crystals. Therefore, the presence of the used additive is an important CPP regarding product quality.

### Characterization of Crystal Size, Habit, and Powder Flowability

The polymorphism of the crystalline product depended on the presence of dissolved PVP-K12 in the crystallizing solution. The two polymorphs have different crystal habits. The kinetically favored Form B exhibits needlelike crystals, while the thermodynamically stable Form A is characterized by an isometric shape. The difference in the crystal habit is the primary source of varying powder rheological properties.

**Figure 6:**
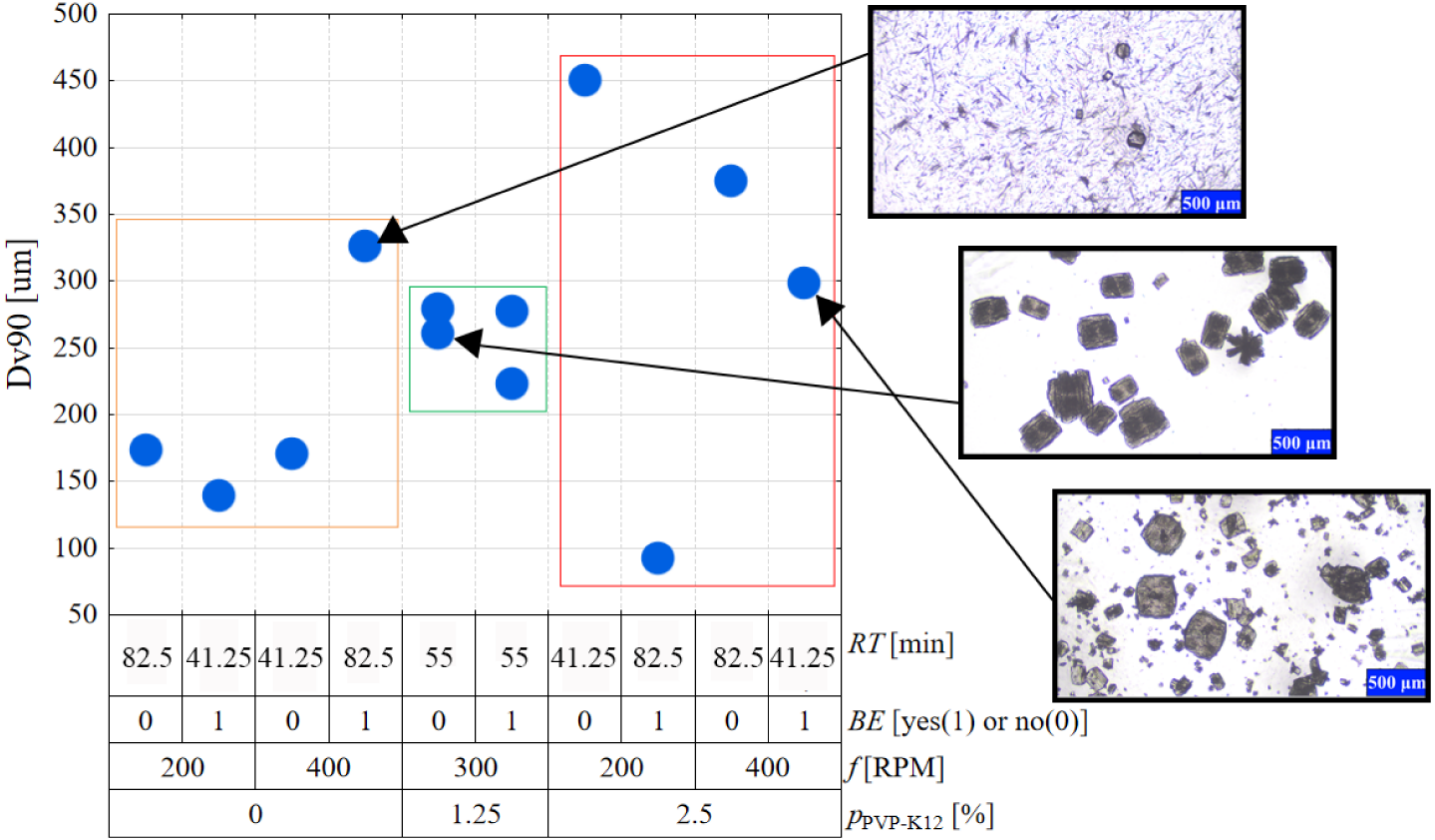
Variability plot of Dv90 as a function of the four examined process parameters.

In Table 5, some microscopic images and CSD curves of different products can be seen to visualize the effect of the added PVP-K12 polymer additive on polymorphism and crystal size. In the case of FMT_8, the kinetically preferred, needlelike Form B polymorph emerged mostly since no polymer additive was used during crystallization. In the other two experiments listed in Table 5, namely, FMT_11 and FMT_4, 1.25 and 2.5 w/wFMT% PVP-K12 were added to the crystallizing solution, respectively. In all two cases, the isometric crystals of the Form A polymorph could be observed. Comparing the microscopic images, CSD curves and Dv values of experiments FMT_11 and FMT_4 in Table 5 show that increasing the amount of the added PVP-K12 increases the average crystal size, but at 2.5 w/w_FMT_% PVP-K12 concentration, a smaller crystal fraction can be observed on the microscopic images as well. This concludes that the main factor influencing polymorphism is the presence of the additive, while its increasing amount slightly raises inhomogeneity in the final CSD. Examination of the results listed in Table 6 indicates that the intensified mixing conditions (presence of BE and 400 rpm mixing rate) can further alter the CSD curve of the Form A crystals produced with 2.5 w/w_FMT_% PVP-K12, compared to the experiments summarized in Table 5, which were conducted in relatively gentle mixing conditions (no BE and 200 or 300 rpm mixing rate). The formation of smaller crystals can occur due to the inhibited crystal growth caused by intensive shear forces in the crystallization medium, which is aided by vigorous agitation. Based on this idea, the factor interaction of BE and mixing rate becomes more significant on the final crystal size profile of the product as the PVP concentration increases. Nevertheless, the applied fractional factorial design is not suited to precisely examine possible factor interactions since they confound with one another. However, this trend can be well followed on the variability plot of the Dv90 values in Figure 6. When no polymer is present in the initial solution, mostly the needlelike Form B polymorph crystallizes, whose CSD is less sensible for other process parameters. The explanation for the outlier is the increased presence of larger Form A particles compared to the other products prepared without the additive. When the polymer additive is present, only Form A crystallizes, but the final CSD is primarily dependent on its amount, and the mixing conditions defined by the BE and the mixing rate. Interestingly, no correlation between the applied residence time and crystal size can be discovered. For this reason, it is advisable to fix the amount of PVP at 1.25 w/w_FMT_%, the stirring rate at 300 rpm, and neglect the buffer element to obtain pure Form A particles and to aid representative product withdrawal in continuous crystallization.

**Table 5:**
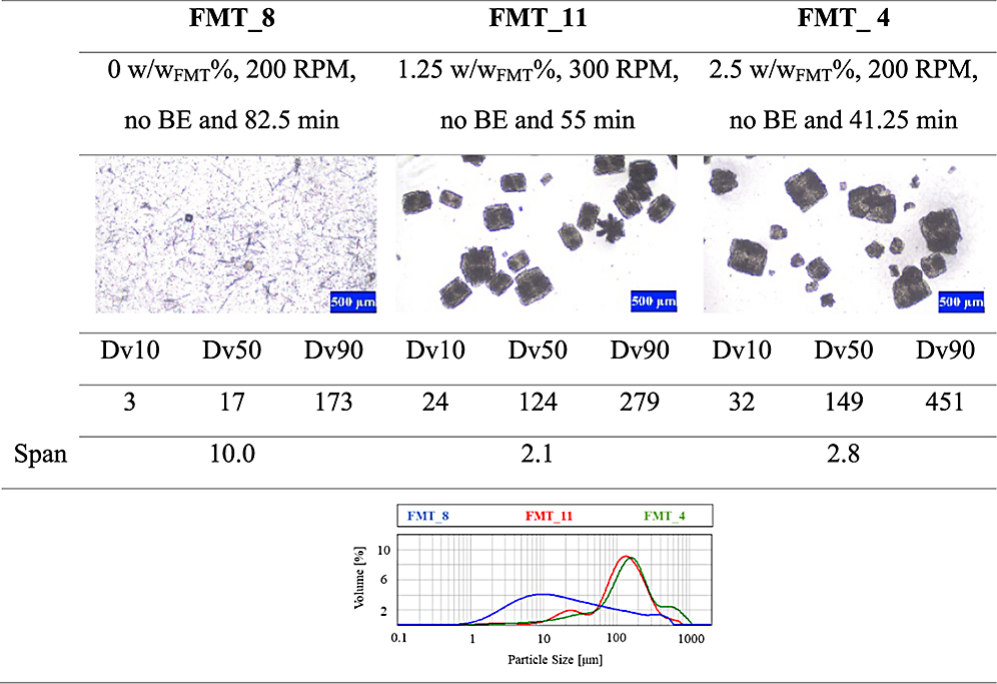
Effect of the Amount of the Added Polymer on Product Polymorphism and Crystal Size^a^

a All Dv values and the span are given in μm.

**Table 6:**
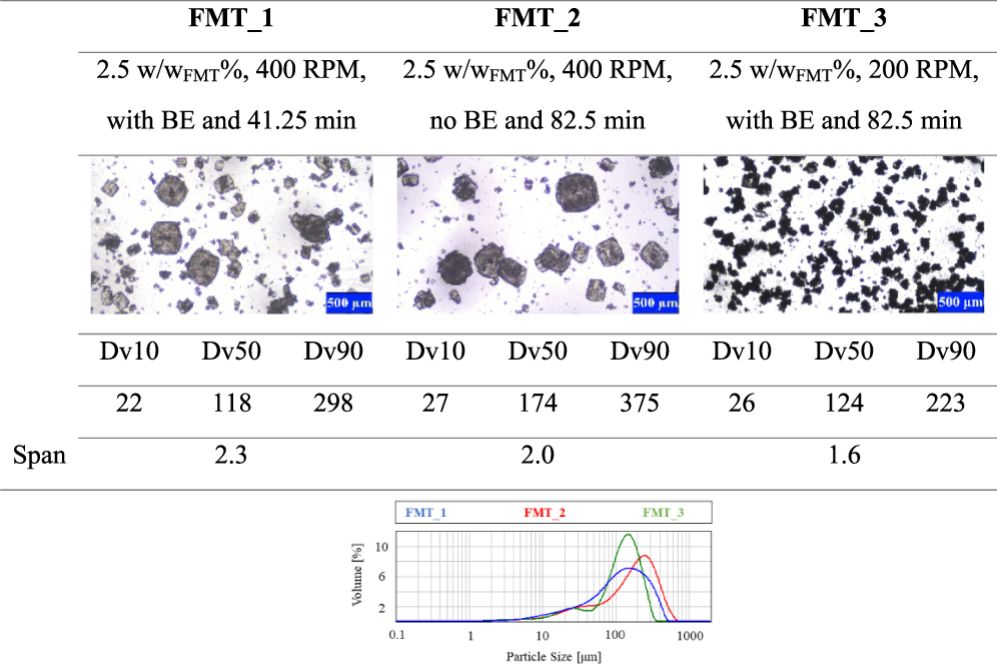
Effect of Mixing Rate and Presence of the Buffer Element on Product Crystal Size^a^

a Dv values and the span are given in μm.

**Figure 7:**
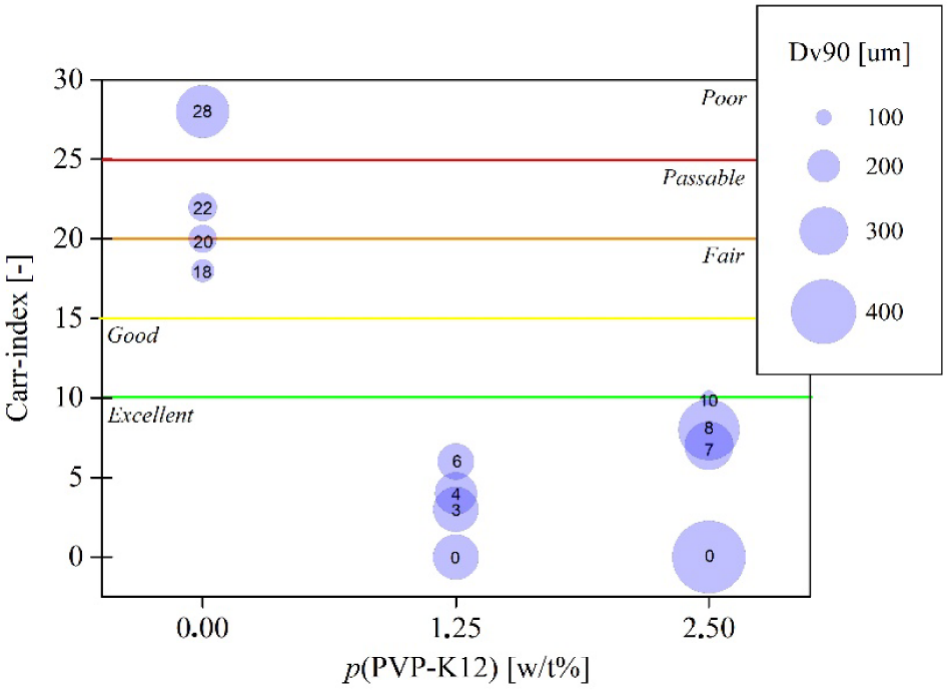
Carr index (the number inside the blue circles) and Dv90 volumetric distribution value (visualized as the diameter of the circles) as a function of *p*_PVP-K12_.

Powder flowability was characterized by calculating the Carr index of each sample. According to Figure 7, it can be concluded that adding PVP-K12 generally enhances the particles powder flowability. It is in correspondence with the previous statements, since PVP-K12 induces the crystallization of the better-flowing Form A polymorph and increases the average particle size. Besides *p*_PVP-K12_, no other process parameter seems to be influential regarding powder flowability. Nevertheless, the amount of the additive affects the size of the forming Form A crystals but also leads to inhomogeneity, which slightly worsens powder flowability (see Figure 7).

In summary of the batch results, the polymer amount is advisable to fix for **1.25 w/w**_**FMT**_**%** in continuous operation, to obtain pure Form A particles with homogeneous crystal size. Higher polymer concentration (2.5 w/w_FMT_%) yields Form A crystals as well, but inhomogeneity in size increases, which show greater dependence on mixing characteristics (i.e., stirring rate and *BE*) and increases fluctuation in powder flowability as well. Mixing rate should be kept at a lower value as far as possible, as it might intensify the formation of small crystals. However, regarding representative and steady product withdrawal, it should be fixed at such a value that hinders the sedimentation of the formed crystals. For these reasons, the mixing rate was fixed at **300 rpm** in continuous crystallization experiments. The buffer element on its own did not facilitate inhomogeneity in crystal size; however, its presence increased the deviation in yield. Therefore, **no** BE was applied in the continuous crystallization system. In terms of maximizing production, a lower dosing rate is recommended; therefore, *FR* was set to **10 mL/min**.

### Continuous Crystallization

The previously described preliminary batch experiments were fundamental to determine the process parameters for continuous additive-controlled crystallization. Analysis of the four examined process parameters (*p*_PVP-K12_, *RT* or *FR*, BE, and *f*) revealed that polymorphism-wise pure and well-flowing crystalline particles can be produced by the dissolution of 1.25 w/w_FMT_% PVP-K12 in the FMT feed solution. Mixing rate was set to 300 rpm in all four MSMPR units to achieve steady homogeneous product withdrawal without negatively affecting the crystal size. Moreover, some process parameters were fixed in advance, i.e., FMT concentration (0.007 g/mL water solution), initial solution temperature (60 °C), and temperature steps (30–20–10 °C). Considering the results of the preliminary batch experiments, two scenarios of continuous crystallization were tested in depth to clearly determine the effect of additive: one without the additive and one with 1.25 w/w_FMT_% PVP-K12 added to the crystallizing solution. The set process parameters of the three-stage MSMPR system and the main results are summarized in Table 7.

**Table 7:** Results of the Continuous Crystallization Experiments

exp. name	*p*_PVP-__K12_[w/w_FMT_%]	*y* [%]	polymorphism
FMT_C_1	0	70.8	mixture of Form A and Form B^a^ (av. 0.5% Form A)
FMT_C_2	1.25	71.1	Form A

a Polymorphic composition was determined by Raman spectroscopy mapping and CLS evaluation of the samples collected at different residence times.

### Continuous Crystallization without Additive

When no additive was present in the FMT solution, the mixture of kinetically preferred Form B and thermodynamically stable Form A polymorphs crystallized. This phenomenon continued through the entire crystallization process and could be well followed on the microscopic images taken of the samples collected at every *RT* (see Table 8). The CSD curves of the samples can be described as bimodal. The second smaller hump can be explained by the aggregation of the needlelike crystals of FMT Form B. FMT Form A was present in the product samples from the first residence time, but its amount remained constant (0.5%) throughout the whole time. Based on the shift of the peak of the main crystal fraction from 10 to 20 μm, and the Form A content, the system reached steady state by the fourth residence time. The stable operation of the system came against difficulties regarding clogging due to the needlelike habit of FMT Form B. The water, in which the formed product crystals were carried as a suspension due to its strong surface tension, tended to build up in the horizontal overflow tube of the third MSMPR unit. This disadvantageous phenomenon could be overcome by inserting a thin copper rod inside the overflow tube and its rubber tube extension, which ensured that water continuously flowed through this transfer zone. According to the evolution of yield displayed in Table 8, it has a run-up period as well, and it becomes more or less stable from the fourth residence time. At the third residence time, an outlier yield value can be identified. The explanation for this sudden increase in yield is that while the flow of water was significantly enhanced with the copper rod inserted in the transfer zones, the needlelike crystals still tended to stick in the overflow tubes. This allowed a small amount of suspension to build up in the overflow tube and caused a noticeable deviation in the yield. The overall yield was 70.8%, and the productivity turned up to 2.97 g/h, but the product was poor flowing.

**Table 8:**
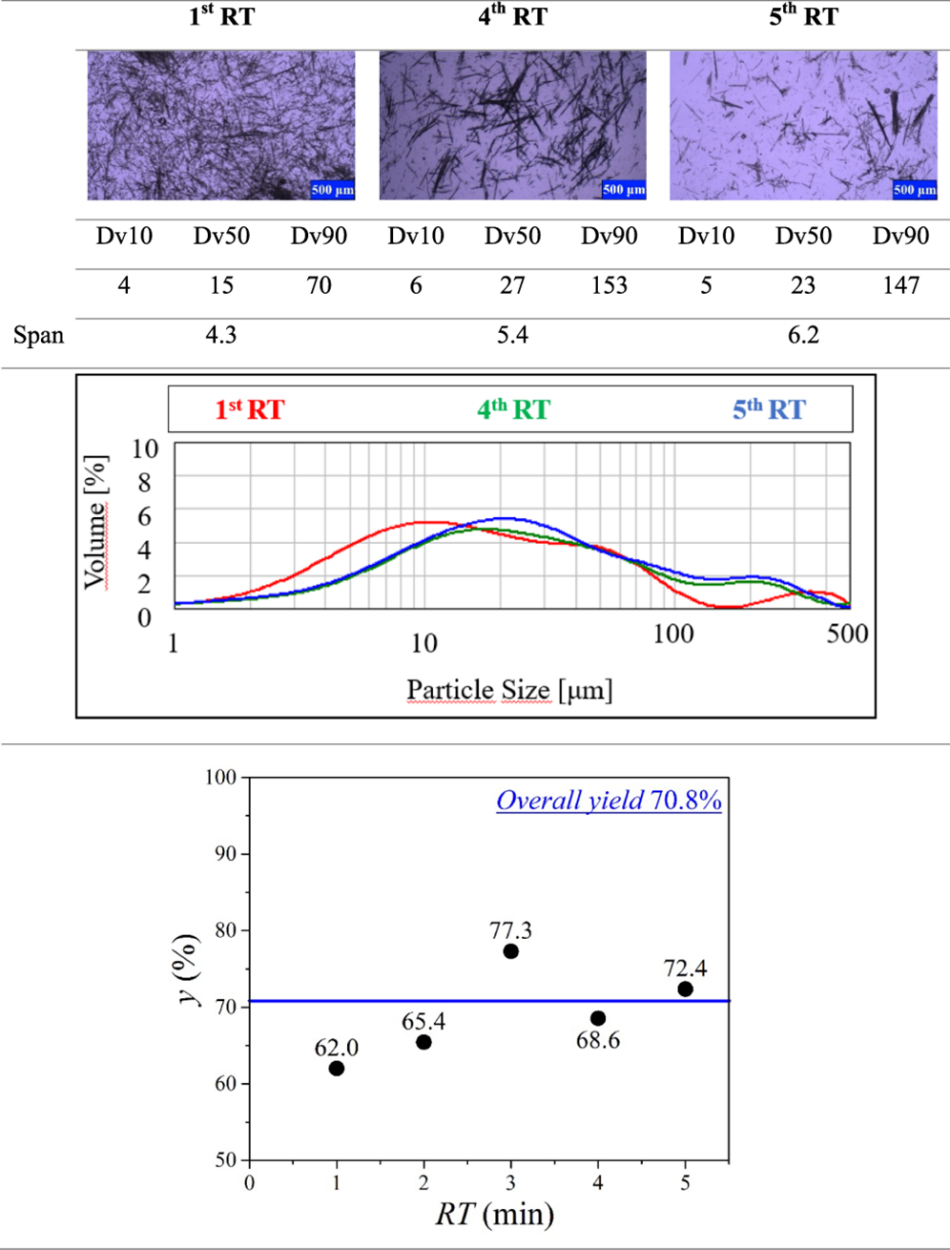
Evolution of Crystal Size, CSD, and Yield during the Continuous Crystallization without Additive^a^

a All Dv values and the span are given in μm.

### Continuous Crystallization with Additive

Continuous crystallization of FMT was accomplished in the presence of 1.25 wt %_FMT_% PVP-K12 as well. In contrast to the process without any additive, the stable Form A polymorph crystallized from the beginning and the Form B polymorph did not appear at all. The product had excellent flowability, and it was free from any remaining polymer (see the Supporting Information). The system could be operated for more than 5 *RT* (>6.5 h) without any difficulties or clogging. Considering crystal size, the CSD curves, Dv values, and optical microscopic images of different samples collected at succeeding *RT*’s indicate that the system reached the steady state for the third *RT* (Table 9), one residence time earlier than without the additive. The overall yield was 71.1% close to the achieved yield in the absence of the additive, while the productivity increased to 2.99 g/h, which is a 4-fold improvement compared to the batch results. Looking at the consecutive yield values in Table 9, it can be concluded that the fluctuation in yield was smaller than without the polymer additive and stabilized from the third residence time. The results of the continuous experiments affirm that, to achieve pure, homogeneous, and well-flowing Form A crystals, PVP-K12 must be added initially to the crystallizing solution. The formation of the isometric Form A polymorph also aided the stable operation of the system, as clogging did not occur at all. In conclusion, FMT Form A could be selectively crystallized by adding 1.25 w/w_FMT_% PVP K-12 formulation additive to the crystallizing solution initially in a three-stage MSMPR cascade. Compared to the continuous crystallization without the additive, the process had a good yield and a shorter run-up period. The produced Form A crystals had excellent powder flowability characteristics, which enabled the stable operability of the system as no clogging occurred.

**Table 9:**
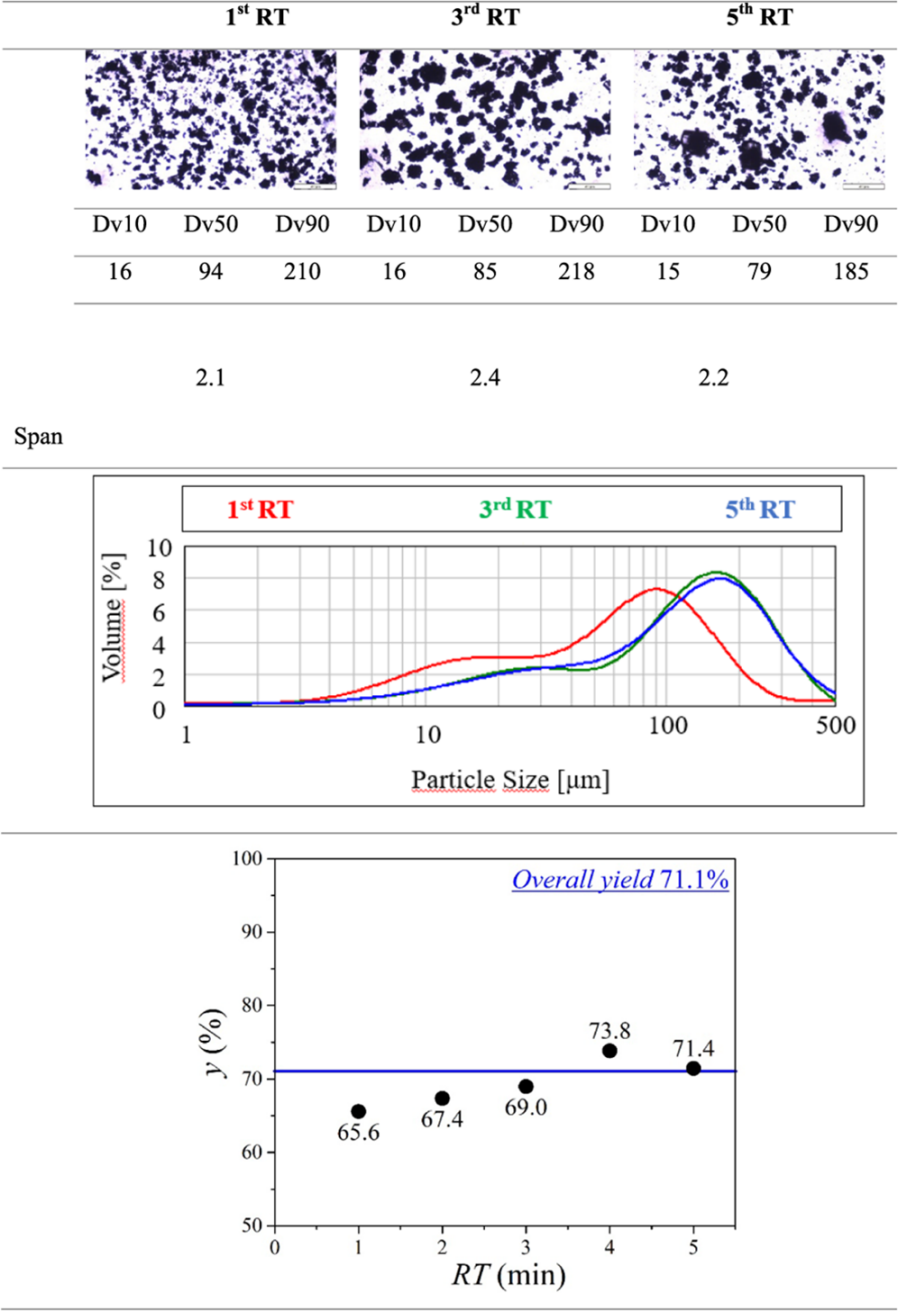
Optical Microscopic Images and CSD Curves of the 1.25 w/w_FMT_% Additive-Controlled Continuous Crystallization Samples at the First, Third, and Fifth RT^a^

a Dv values and the span are given in μm.

## Conclusions

In this work, a novel process using a three-stage MSMPR cascade crystallizer was developed for the continuous additive-controlled crystallization of famotidine with PVP-K12. The aim was to present a systematic workflow on identifying CPPs of a continuously operated additive-controlled crystallization process, since the number of relevant studies is under-represented in the literature. These crystallization processes can utilize both the advantages of continuous manufacturing (steady-state operation, constant product quality, etc.) and additive effects (crystal size, habit, polymorphism control, minimizing fouling, etc.).

Altogether, seven CPPs were examined on key CQAs (yield, polymorphism, morphology, and powder flowability), and the continuous operability was evaluated. Beforehand, three of the seven CPPs (*c*_FMT_, *T*, and temperature step profile), regarding continuous operability, were fixed based on empirical observations. The remaining four CPPs (*RT* – *FR*, *p*_PVP-K12_, *f*, and BE) were examined in depth by applying 2^4–1^ fractional factorial design. It was found that the yield depends on the set *RT* or its continuous equivalent *FR* and the presence of buffer element (BE). For higher yields, longer *RT* (slower *FR*) should be applied. The buffer element in our case did not improve mixing efficiency as expected and described in other publications but increased the standard deviation of yield. Therefore, 10 mL/min of *FR* and no BE were set in continuous mode. The presence of PVP-K12 promoted the nucleation of FMT Form A; thus, the product crystals comprised only the thermodynamically stable Form A polymorph. In contrast, the product was always a mixture of the kinetically preferred Form B and Form A without the additive. Since Form A exhibits isometric crystals, its powder rheological properties are better than the needlelike Form B. Increasing the amount of the polymer additive enhanced crystal growth but combined with other factors caused an overall inhomogeneous CSD. Besides this phenomenon, constant suspension flow and representative product withdrawal in continuous operation had to be taken into consideration. Due to these listed aspects of mixing characteristics, generally 300 rpm mixing rate was set in the MSMPR units.

With the CPPS specified, two continuous crystallization experiments were conducted and analyzed, one without the additive to serve as a blank run and one with 1.25 w/w_FMT_% PVP-K12. As could be expected based on batch experiments, continuous crystallization without the polymer resulted in a mixture of Form A and Form B. In contrast, when 1.25 w/w_FMT_% PVP-K12 was initially added to the crystallization solution, the thermodynamically stable Form A crystallized from the beginning. The yield (71.1%) remained stable throughout the process, no trace amounts of Form B or PVP could be detected, and the flowability was excellent.

The developed additive-controlled continuous crystallization in a three-stage MSMPR cascade crystallizer offers the stable production of famotidine Form A. The achieved 2.99 g/h productivity is 4.7 times greater than that in the corresponding batch run. The workflow, construction, and statistical analysis of the preliminary experimentation process can give a possible example for developing other continuous additive-controlled crystallization methods. These findings also lead to the conclusion that the fundamentum of a continuous additive-controlled crystallization system could be successfully determined by shorter batch experiments and Design of Experiment methods. Future work should focus on the optimization of the assembled system to reach a higher yield.

## Supporting Information Available

Examination of the effect of the amount of PVP-K12 additive on famotidine morphology and quality, different powder flowability classes based on the Carr index, examination of product PVP-K12 content by Raman-mapping, and residuum analysis of the fitted statistical model (PDF)

## Abbreviation Legend

*Abbreviation*: *Term*
*C*_solution_: concentration of the famotidine solution
α: statistical significance level
API: active pharmaceutical ingredient
BE: buffer element
CCD: charge coupled device
CLS: classical least-squares
CPP: critical process parameter
CQA: critical quality attribute
CSD: crystal size distribution
DoE: design of experiment
Dv: volumetric distribution value
*f*: mixing rate
FMT: famotidine
*FR*: flow rate
HPMC: hydroxypropyl methylcellulose
ID: inner diameter
*m*_Form-B_: mass of the measured famotidine Form B
*m*_gross_: mass of the product and glass filter
*m*_*tare*_: mass of the glass filter
∑*m*_*retrieved*_: mass of the total product retrieved
MSMPR: mixed suspension mixed product removal
*p*: statistical p-value
*P*: productivity
PAT: process analytical technology
PLA: poly(lactic acid)
PLC: programmable logic controller
PTFE: polytetrafluoroethylene
PVA: poly(vinyl alcohol)
PVP-K12: poly(vinylpyrrolidone)
*R*: statistical resolution
*RT*: residence time
SAM: self-assembling monolayer
*T*: temperature
TC: tubular crystallizer
*t*_elapsed_: elapsed time
*Var*(*y*): variance
*V*_filling_: filling volume
*y*: yield
