# Supplementary material for: Development of Continuous Additive-Controlled MSMPR Crystallization by DoE-Based Batch Experiments
Source: Ind Eng Chem Res. 2024 Jul 23;63(31):13709–22. doi: 10.1021/acs.iecr.4c01933 (PMC11311132; doi:10.1021/acs.iecr.4c01933)
Supplement: Supplementary file 1 — ie4c01933_si_001.pdf [file ie4c01933_si_001.pdf]

Supplementary Information for

Development of continuous additive-controlled MSMPR crystallization by DoE based batch experiments

György Nimród Stoffán[1], Zsolt Lőrincz[1], Éva Pusztai[2], Lajos Madarász[1], Kornélia Tacsi[1], György Marosi[1], Hajnalka Pataki[1]*

[1]Department of Organic Chemistry and Technology, Faculty of Chemical Technology and Biotechnology, Budapest University of Technology and Economics, Műegyetem rkp. 3., H-1111 Budapest, Hungary

[2]Department of Chemical and Environmental Process Engineering, Faculty of Chemical Technology and Biotechnology, Budapest University of Technology and Economics, Műegyetem rkp. 3., H-1111 Budapest, Hungary

Corresponding author: Hajnalka Pataki

e-mail: pataki.hajnalka@vbk.bme.hu

Examination of the effect of PVP-K12

Before conducting the preliminary batch experiments, the effect of PVP-K12 on FMT's morphology was examined. The experiments were carried out in a PLC-controlled crystallizer (see Figure 1 in the article). For every experiment 70 ml of 44.3 mg/ml aqueous FMT solution, 300 RPM mixing rate and 0.6 °C/min cooling rate from 90 °C to 25 °C were applied to exclude interplay with $p_{\text{PVP-K12}}$. The screened $p_{\text{PVP-K12}}$ amounts were 10, 5, 2.5 and 1 w/t% respectively. The results of the experiments are summarized in Table S1. After crystallization, the suspension was filtered through a G3 porosity glass filter with a membrane pump and air dried for 3 days. Subsequently, the maximum production was calculated from the weighed mass (see Eq. 2. in the article) and the physical and chemical properties of the product were determined by different analytical methods (see "Experimental materials and methods" chapter in the article). Since 10

and 5 w/t% PVP-K12 caused the forming solid product to stick and coat the glass crystallizer wall, the upper value of $p_{\text{PVP-K12}}$ was fixed at 2.5 w/t% in the fractional factorial design.

Table S1. – Preliminary experiments on the amount of added PVP-K12

**exp. name**	$p_{\text{PVP-K12}}$ **[w/t%]**	**Results**
pre_FMT_ 1	10	The product coated and stuck on the crystallizer wall. The liquid phase did not contain any crystals at all, and the solid coating was very hard to work up.
pre_FMT_ 2	5	Similar to experiment pre_FMT_ 1.
pre_FMT_ 3	2.5	Homogenous solid particles in the suspension and less crystals stuck on the crystallizer wall. Pure Form A.
pre_FMT_ 4	1	Similar to experiment pre_FMT_3.

Powder flowability classes

In Table S2 the powder flowability classes which were used to characterize each experiment's product are summarized according to the Carr-index.

Table S2 – Powder flowability classes

**Carr-index**	**Powder Flowability**
≤ 10	excellent
11 – 15	good
16 – 20	fair
21 – 25	passable
26 – 31	poor flow
32 – 37	very poor flow
≥ 38	approximately no flow

Product PVP-K12 content examination

The remaining PVP-K12 content of the continuous crystallization product was examined with Raman mapping. A total of 6561 single measurement points per map was an efficient method to detect trace amounts (0.5 w/t%) of solid PVP-K12 dispersed amongst FMT crystals. The spectra were normalized, and linear baseline correction was applied. To enhance the limit of detection in the case of PVP-K12 analysis, the normalized intensity of the reference FMT Form A's and PVP-K12's spectra were adjusted according to their Raman activity. This adjustment was based on the ratio of FMT Form A's peak at 545.6 cm^{-1} and PVP-K12's peak at 933.3 cm^{-}

[1]. The two reference spectra were recorded with the same settings (40 sec spectral acquisition time per spectrum, 2 accumulation number) to maximize the intensity of the peaks and to make them comparable. Examining the additive assisted (1.25 w/t% PVP-K12) continuous crystallization experiment's product, it can be concluded that the sample either contained less than 0.5 w/t% or no remaining PVP-K12 at all.

Residuum analysis

Batch experiments – Statistical analysis of yield

To check the adequacy of the statistical analysis' results of the fractional factorial design, the residuals were examined. Plotting residuals against case numbers, it can be concluded that randomizing the order of the experiments ensured the independency of errors, as no trend can be seen in the varying of their values (see Figure S1).

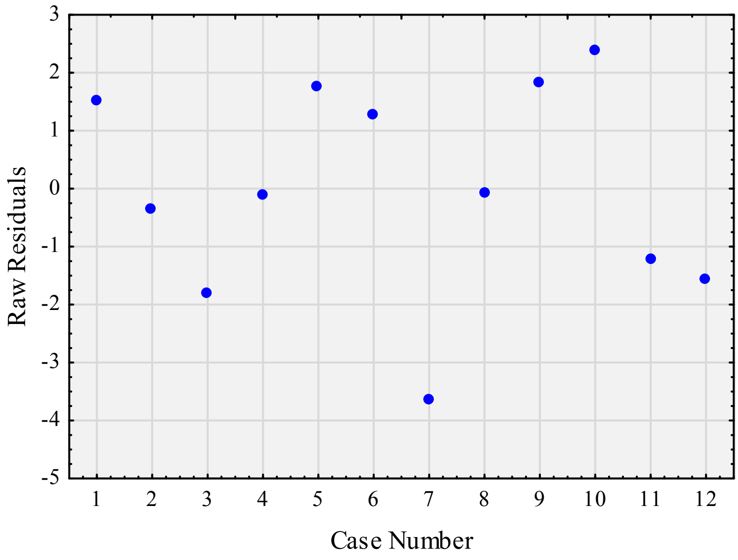

Figure S1. - Residuals vs. case numbers

As statistical analysis revealed, residence time (*RT*, or its continuous equivalent flow rate – *FR*), and the presence of the buffer element (*BE*) proved to be the statistically significant factors affecting yield. However, plotting the observed yield values against *RT* and *BE* shows the difference of functionality, that is if *BE* is absent from the crystallizer, then yield is a sigmoid function of *RT*, while when *BE* is present, then yield changes exponentially (see Figure 4b in the article). Therefore, the analysis of the residuals was done separately for the two scenarios.

Nevertheless, the repetition of the center point experiments resulted small standard deviation in yield, which indicate that the experiments are repeatable, and it is independent of *BE*. Plotting the predicted yield values (calculated from the fitted equations) against the observed ones, shows that with the *BE* present at higher yield values, the residuals increase, as the observed yield's standard deviation increase too (see Figure S2a and S2b). This affirms that yield follows an exponential like functionality of *RT*. However, the number of residuals available might be a limiting factor of the precise evaluation. Nevertheless, based on the normal plot, the errors seem normally distributed as the expected normal values follow a linear trend (see Figure S).

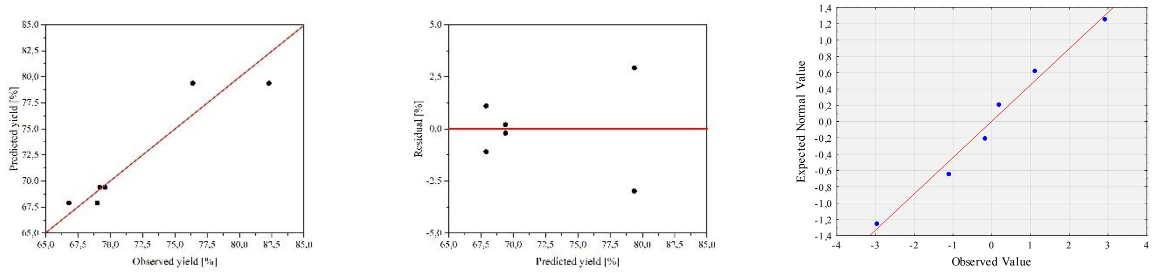

(a) **(b)** **(c)**

Figure S2. – Predicted vs. observed yield values **(a)**, residuals vs. predicted yield values **(b)** and normal plot **(c)** in the presence of *BE*

If the buffer element was not present during crystallization, a sigmoid function describes yield as a function of *RT*, which is affirmed by the repeated center point experiments. In this case, the fitted sigmoid model is an adequate approximation (see Figure S3a), the errors are of constant variance (see Figure S3b) and approximately normally distributed (see Figure S3c).

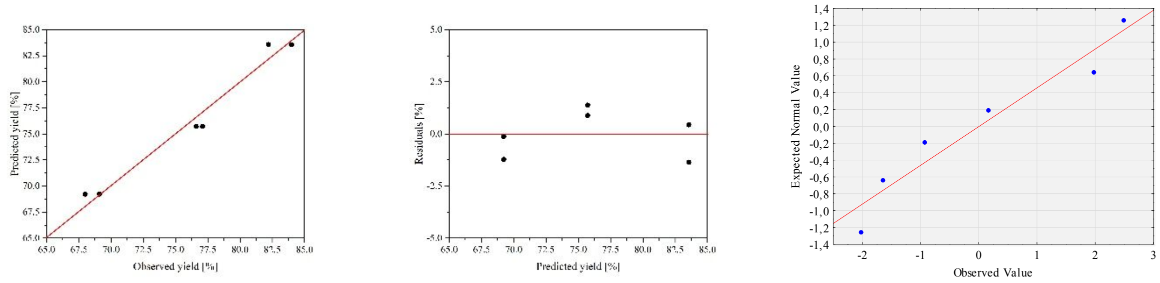

(a) **(b)** **(c)**

Figure S3. - Predicted vs. observed yield values **(a)**, residuals vs. predicted yield values **(b)** and normal plot **(c)** in the absence of *BE*
